# Nurses’ Perspectives on Cancer Care for People Who Experience Incarceration in British Columbia, Canada: Challenges and Opportunities

**DOI:** 10.1177/08445621261449582

**Published:** 2026-05-12

**Authors:** Mar’yana Fisher, Leah K. Lambert, Olga Petrovskaya, Helen Brown, Kelli I. Stajduhar

**Affiliations:** 1 School of Nursing, 8205 University of Victoria, Victoria, Canada; 2 Nursing and Allied Health Research and Knowledge Translation, 8144 BC Cancer, Vancouver, Canada; 3 School of Nursing, University of British Columbia, Vancouver, Canada; 4 School of Nursing, 8205 University of Victoria, Victoria, Canada; 5 School of Nursing, 8205 University of Victoria, Institute on Aging & Lifelong Health, Victoria, Canada

**Keywords:** cancer, cancer treatment access, incarcerated populations, health systems research, interpretive description, Actor-network theory

## Abstract

Cancer is the leading cause of death for Canadians. Research by Canadian scholars has highlighted that people who experience multiple and intersecting socio-economic barriers are more likely to receive a diagnosis only after cancer has become advanced. For people who experience incarceration, structural barriers to cancer treatment and care are further exacerbated. Yet, few studies in Canada have focused specifically on the provision of cancer care in provincial correctional settings. This study explored how nurses understand and navigate the delivery of cancer care to people who are incarcerated in British Columbia. Guided by Interpretive Description (ID) and informed by actor-network theory (ANT), the research aimed to generate practice-relevant insights by examining nurses’ perspectives, experiences, and the systemic factors shaping their clinical realities. Findings from this study illustrate nurses’ views that the prison's focus on security shapes how, when, and whether cancer care is delivered. Drawing on interviews with nurses, the analysis produced the following insights: 1) institutional priorities often complicated the delivery of cancer care; 2) nurses perceived systemic stigma and institutional mistrust toward patients who are incarcerated; and 3) lack of confidentiality during healthcare encounters hindered patient-centred cancer care. Despite this, nurses frequently found ways to navigate the professional tensions arising from dual responsibilities to patient care and incarceration. These insights show how cancer care is often contingent on nurses’ ability to navigate systemic constraints and relational barriers, underscoring the need for integrated, trauma-informed, and equity-oriented approaches that support continuity of care across correctional and healthcare systems.

## Introduction

Cancer is the number one cause of death for Canadians, with the number of cases rising annually due to population growth and aging (Canadian Cancer Statistics [CCS], 2023). Despite the overall reduction in cancer incidence and progress in cancer prevention, treatment, and screening, an estimated 88,100 people were expected to die from cancer in 2024 (Brenner et al., 2024), with 96% of these deaths projected to occur among individuals 50 years of age and older (CCS, 2023). As national cancer data undergo rigorous collection and verification processes, final figures for 2024 will not be available until the next report is released in 2026 (Brenner et al., 2024). While these reports offer important insights into cancer prevalence and mortality trends, they largely reflect population-level factors such as age, sex and behavior (Brenner et al., 2024). As a result, Canadian cancer data does not yet account for contextual influences and social determinants of health (Brenner et al., 2024), obscuring correlations between cancer burden and structural inequities. Specifically, research by Canadian scholars (Bourgeois et al., 2023; Lambert et al., 2023) has highlighted that people who experience multiple and intersecting socio-economic barriers to accessing healthcare, such as discrimination, poverty, and insecure housing, among others, are more likely to receive a diagnosis after their cancer has become advanced. In addition, individuals facing these barriers often encounter difficulties accessing cancer prevention and treatment services, which contributes to their limited inclusion in broader cancer control efforts (Lambert et al., 2023; Salas et al., 2024). For people who experience incarceration, structural barriers to cancer treatment and care are further exacerbated (Puglisi et al., 2021). Cancer mortality is higher among people who experience incarceration than in the general population, and the mortality risk persists post-release (Oladeru et al., 2022). Population-based data from Ontario show higher cancer mortality among people who experienced incarceration, with standardized mortality ratios of 1.6 for men and 1.4 for women compared with the general population (Kouyoumdjian et al., 2017). In the US, a longitudinal cohort study using data from 690 individuals in Connecticut found that among individuals diagnosed with local or regional cancer, adherence to recommended treatment measures was lower for individuals diagnosed during incarceration (49.2%) than for those without an incarceration history, who received 66.7% of recommended care (Oladeru et al., 2025). However, these challenges are not coincidental but are actively produced and reinforced through complex and intersecting practices of healthcare and carceral systems. In concert, healthcare organizations and correctional institutions, both tasked with maintaining health and safety, contribute to the ongoing disparities in cancer care (Armes et al., 2024; Oladeru et al., 2022).

In 2023, Canada's total incarcerated adult population was 34,986 (World Prison Brief, n.d.), with an average of 1,712 adults held across 10 provincial correctional centers in British Columbia (Statistics Canada, 2025). Despite these incarceration rates, there is limited evidence about access to and the quality of cancer care for people who experience incarceration (Kouyoumdjian et al., 2017). Considering the significant population cancer prevalence and disparities in access to cancer care for people who experience incarceration, it is crucial to explore how healthcare providers understand and navigate the delivery of cancer services for this population.

Evidence on how access to cancer diagnosis and treatment is shaped within correctional settings remains limited in the Canadian context, despite emerging findings from other countries. For example, Armes et al. (2024) conducted interviews with people in prison, correctional staff, healthcare providers, and oncology specialists in England. Key barriers to timely cancer diagnosis and treatment included communication breakdowns, logistical challenges, and limited patient autonomy. In contrast, factors such as the recognition of cancer-specific symptoms and the role of peer advocacy, were noted as important enablers of access to cancer care.

In Canada, Kouyoumdjian et al. (2017) examined healthcare access among federally incarcerated individuals in Ontario. The authors found that while participants in this study often had greater access to primary care than in the community, significant barriers remained, particularly for specialized services such as cancer care. Although national data on cancer in the prison population is limited, available evidence suggests that cancer risk factors are overrepresented among incarcerated populations (Kouyoumdjian et al., 2017). In Ontario, a population-based study reported a 10-year cancer prevalence of 0.4% in men and 0.6% in women at the time of admission to provincial custody in 2000 (Kouyoumdjian et al., 2017). Between 2000 and 2012, cancer incidence was 2.6% for men and 2.8% for women (Kouyoumdjian et al., 2017). While the overall cancer incidence was similar to the general population, the younger age profile of individuals in this study, higher rates of cancer mortality and elevated incidence of specific cancers such as lung, cervical and liver are concerning. These factors might reflect inequities in diagnosis, treatment and access to cancer care for people who experience incarceration.

Few studies in Canada have explicitly focused on cancer care for people in provincial custody, where the organization of healthcare delivery differs from federal correctional settings and may present unique challenges and opportunities. For example, in British Columbia (BC), the Provincial Health Services Authority (PHSA) is responsible for providing healthcare in provincial jails, a mandate for health service delivery that is generally seen as more integrated and of improved quality than federally delivered correctional healthcare (McLeod et al., 2024).

Healthcare for people in British Columbia's correctional facilities is delivered through on-site correctional health services that provide primary, mental health, and substance use care delivered by multidisciplinary teams (BC Mental Health & Substance Use Services [BCMHSUS], 2025). Individuals experiencing health concerns are typically first assessed by the facility's healthcare team, which manages and coordinates referrals to external specialists or hospital-based services when advanced diagnostics or treatment are required (BCMHSUS, 2025). Provincial correctional facilities do not provide inpatient or specialized oncology services; as a result, cancer diagnosis and treatment generally occur in community or hospital settings outside the facility. Accessing these services requires coordination across health and correctional systems, including transportation and security arrangements, which can introduce delays, limit patient autonomy, and disrupt continuity of care. Thus, understanding how cancer care is delivered within these distinct settings is crucial for informing future practice, research, and policy.

## Purpose and Research Questions

The purpose of the research study was to explore how nurses understand and navigate the delivery of cancer care for people who experience incarceration in the Canadian province of British Columbia. The research questions were: How do nurses describe the delivery of cancer care for people who experience incarceration in British Columbia? How do nurses perceive and navigate the systems, clinical practices and relationships involved in providing cancer care to people who experience incarceration? What challenges and opportunities do nurses identify in cancer care services for this population?

Throughout this paper, we use the term “people who experience incarceration” to refer to individuals currently or formerly held in provincial correctional facilities. This term is used to reflect a person-centered, non-stigmatizing approach that acknowledges the broader social and structural contexts of incarceration. This term is consistent with best practices in correctional health and public health research (Bedell et al., 2018).

## Methods

### Study Design

This study was guided by Interpretive Description (ID), a qualitative methodology designed to generate rich, practice-relevant insights into health-related experiences (Thorne, 2016; Thorne et al., 1997). ID supports the exploration of complex clinical phenomena in context, making it especially well-suited for research grounded in nursing practice. In this study, the ID design supported an in-depth description of nurses’ perspectives on delivering cancer care to individuals who experienced incarceration. It focused attention on nursing experiences and systemic factors that shaped their clinical realities, with the aim of generating practice-relevant insights. Additionally, the underpinnings of ID align well with actor-network theory (ANT), informing this study.

### Theoretical Perspective

This study was informed by actor-network theory (ANT), which offers a constellation of sensitizing concepts and theoretical approaches that draw attention to the complexity of networks involving both human and nonhuman actors, including physical environments and documentation (Nimmo, 2011). Rather than providing prescriptive guidance, sensitizing concepts (e.g., relational networks of heterogeneous actors) invite the researcher to flexibly employ them. In this study, ANT helped focus the analysis on how cancer care was co-produced through dynamic interactions among nurses, patients, institutional practices, and various material elements. ANT shaped our focus on relationality, the notion that entities such as people, objects and environments do not exist independently but gain meaning and agency through their interactions (Cresswell et al., 2010; Petrovskaya, 2023). This relational lens, along with ANT's emphasis on analytical symmetry, the idea that people and objects are equally significant in shaping social processes (Law, 1992; Mol & Mesman, 1996), informed the analytic process by guiding attention to material practices that nurses enact and engage in as they navigate and negotiate care across healthcare and carceral systems. For a more detailed discussion of this theoretical perspective, see Fisher et al. (2024).

### Sampling and Recruitment

Ethics approval was obtained from the University of British Columbia BC Cancer Research Ethics Board and the University of Victoria Research Ethics Board prior to commencing the study (protocol number: H23-03774). Participants were recruited through purposive (Thorne et al., 2004) and snowball sampling (Palinkas et al., 2015; Vasileiou et al., 2018) by leveraging the research team's professional and clinical networks relevant to cancer care in British Columbia. Nurses were eligible to participate if they were 19 years of age or older, English-speaking, capable of providing informed consent and had current or prior professional experience with direct clinical involvement in cancer care delivery, as well as those who supported coordination, referral, assessment, or follow-up cancer care for patients transferred from provincial correctional facilities to community oncology clinics in British Columbia.

Recruitment focused on nurses working in community-based oncology services, and experience working directly within correctional institutions was not an inclusion criterion. However, several participants described having previous employment in provincial correctional settings and drew on those experiences when reflecting on cancer care. This variation in professional background was not planned but provided additional contextual insight into how cancer care is navigated across correctional and community settings.

### Participants

The sample consisted of 11 healthcare providers with educational backgrounds in nursing, including nine community-based providers (eight Registered Nurses and one Licensed Practical Nurse) and two nurses in senior leadership roles from the provincial cancer care sector. Participant demographic data did not include biological sex or gender identity; therefore, no assumptions were made about participants’ gender based on their appearance. Participants’ experiences of providing care to people who experience incarceration varied. Six participants described providing cancer care both in community clinics where they currently practice, and in provincial correctional facilities, where they had previously worked (n = 6). Five participants reported providing cancer care exclusively in community-based settings, such as outpatient clinics (n = 5). Overall, six of the eleven study participants had previous professional experience working in provincial correctional facilities.

Five participants practiced in outpatient clinics located near correctional facilities and reported caring for patients transferred from these institutions to receive cancer care. In contrast, other participants described only one or a few instances in which they had witnessed or been directly involved in supporting clients receiving cancer care, either within institutional settings or in the community. Some of these accounts referred to encounters that had taken place years earlier.

### Data Collection

Data were collected by semi-structured interviews in the spring and summer of 2024. The first author (MF) emailed the informed consent form to participants two weeks before the scheduled interviews. When providing signed consent, participants were able to ask questions about the study. The interviewer (MF) confirmed the participants’ understanding of the data collection process and reminded them of their right to withdraw from the study without consequence or to stop the interview for any reason. One-time virtual interviews were conducted using a secure virtual meeting platform and lasted approximately 30–60 min. The interviews were recorded and transcribed into a text document. Interview questions focused on the processes and practices that facilitate cancer access and care for people in provincial correctional facilities. Participants received a $20 e-coffee card for their participation.

### Analysis

Interpretive thematic analysis was conducted to examine nurses’ perspectives on how people who experience incarceration access and receive cancer care services. After listening to interview recordings and reviewing the transcripts for accuracy and data cleaning, initial codes were generated to organize the information. Following data immersion and the development of initial codes, patterns and themes were generated. Themes were treated as tentative, allowing for changes and redefinition as analysis progressed iteratively. The first author (MF) led the data analysis and developed tentative codes, which were refined with close guidance from KS and OP and discussed with all co-authors to ensure the coherence of interpretations and the clarity of themes supported by the data. Informed by the ANT theoretical framework, we approached data analysis with attention to how cancer care practices are shaped through material and relational networks. As themes emerged inductively, we traced how actors, both human (e.g., nurses, patients, correctional staff) and non-human (e.g., shackles, curtains, health forms), interacted to enable or constrain care. While the analysis centered on the nurses’ perspectives, their descriptions of providing care also gave partial insight into how broader networks, including patients, correctional staff and institutional dynamics, are assembled, negotiated, and at times disrupted the delivery of cancer care. In the concluding phases of analysis, we moved toward consolidating themes and developing interpretive explanations that captured the complexities of how, from the nurses’ perspective, cancer care is delivered for people who experience incarceration. The primary researcher engaged in reflexive writing throughout the analytic process to examine how her clinical nursing background and professional assumptions shaped attention to particular aspects of care delivery and system navigation. Consistent with interpretive description, reflexive memo-writing and team discussions were used to surface and question taken-for-granted clinical norms, assumptions about security routines, professional roles, and care pathways within correctional and oncology settings to avoid privileging clinical perspectives over other possible interpretations.

Our research team comprised members who promote equitable healthcare through research and clinical practice, with expertise in cancer and palliative care research (KS/LL), cancer services (LL), experiences of people who have been incarcerated (HB), and actor-network theory (OP). These complementary perspectives supported critical dialogue during analysis, helping to balance clinically grounded interpretations with attention to broader relational and organizational dynamics.

## Results

Three themes were generated: Security as the structuring logic of care in prisons and community, Burden of proof and complex pathways to referral to cancer services, and Tensions and negotiations in cancer care.

### Security as the Structuring Logic of Cancer Care in Prison and Community

Nurses described how security priorities within prison institutions sometimes limited timely or adequate access to cancer care for people who are incarcerated. They highlighted how institutional routines, security protocols and staffing shortages can occasionally create conditions in which cancer care could be deprioritized or delivered under significant constraints. One nurse with previous work experience in a provincial correctional center said that “the priority in prison is making sure meals happen, and medications happen, because those are usually the biggest things that can likely spur a riot… because you are dealing with people's basic human needs,” underscoring how institutional routines are structured around maintaining order and preventing riots. The tasks of medication and food provision are not simply about meeting essential needs; they also function as part of a prison's security strategy. If meals or medications are delayed or disrupted, the facility can destabilize, and unrest can ensue. These routines and other practices, such as movement control, staffing and scheduling of medical appointments, are elements of a broader security apparatus that shape the condition under which cancer care is delivered.

Beyond occasional lockdowns, everyday security protocols can also disrupt access to health services. In some cases, tensions or conflicts between individuals housed in separate units lead to “movement limitations,” restricting some groups from entering the hallway concurrently to avoid potential altercations. Nurse participants shared that in those instances, and during safety lockdowns, they administered medications at a later time by going “door to door, which was significantly more time-consuming.” Participants described how individuals are brought unit by unit, made to line up, present identification, receive medications through a small window and then return to their cells. Other clinical services are suspended during medication rounds, so if anyone needs non-urgent care, they cannot receive it.

In some instances, patient care can be affected by staffing shortages that were identified as a common issue within carceral spaces. One participant shared: “There were times when corrections staffing was so short… that even in an emergency, I was directed not to call an ambulance for a patient because they had no guards to escort him to the hospital.” In addition to correctional staffing shortages, staffing models for members of the healthcare teams, such as nurses and licensed practical nurses, reflect institutional priorities around risk management rather than clinical availability. Provincial jails housing men do not have healthcare staff available after 11 p.m. Clinical coverage ends before midnight in 9 of British Columbia's 10 provincial correctional centers. The only correctional facility with a 24-h nursing staff is the provincial Correctional Center for Women, which offers a mother-child initiative. On occasion, prescription orders from cancer centers would need to be modified to reflect staffing schedules in jail. Unless patients were allowed to self-administer medications, “they wouldn't have their medications overnight because there is no overnight healthcare staff.” Participants discussed how routines, protocols, and staffing issues impact cancer care, as *they matter medically*, not just logistically. This sentiment was candidly summarized by one of the participants:

The safety and security of the prison is number one. The corrections officers run the show, and so, while we can sometimes push for things, especially if it's an emergency…It can be hard, and it can be like pulling teeth sometimes.

Lastly, participants noted that security priorities extended into the scheduling of cancer care appointments. Nurses at cancer clinics commented that guidelines in correctional facilities do not allow them to tell patients when their appointments will be scheduled to prevent “the risk of [patients] meeting somebody, either for a drug drop or escape.” Our data revealed the complexity and contradictions inherent in this process:

The correctional staff and the inmate aren’t allowed to be told anything when they come to the appointment, and when their next appointments are. Everything has to come through the healthcare department, and so we arrange everything and hope that they [patients] don’t get told, but if they are coming for a daily radiation, they know where they’re going.

### Burden of Proof and Complex Pathways to Referral

We sought to understand nurses’ views on how people who experience incarceration access cancer care, beginning with diagnosis and subsequent referral to community cancer clinics. Participants who did not have previous experience working in correctional institutions had a limited understanding of the various steps of the referral process, as one nurse reflected: “My guess would be that maybe prior to them being referred to us, there could be more happening behind the scenes.” However, the same participant described how they observed the referral process in the oncology clinic: “The doctors get the referral…and they look at all the information… and [decide that] this patient needs to be seen within this many days and then they get booked in.” While there is a standard medical referral process between provincial correctional facilities and community cancer clinics, the participants in this study who previously worked in provincial correctional centers explained that access to cancer care depends on two distinct starting points: 1) a pre-existing cancer diagnosis established in the community before incarceration and 2) developing or presenting with worsening symptoms while incarcerated without a prior diagnosis. Several participants also emphasized that it is difficult to describe one “typical” pathway to care; instead, cancer care trajectories unfold on a case-by-case basis and are shaped by various contextual factors, such as the existence of a prior healthcare record, health concerns that are not being taken seriously, and access to outside appointments.

When a person is remanded to a provincial correctional facility, the Correctional Health Services (CHS) team, operating under the BC Mental Health and Substance Use Services (BCMHSUS) branch of the Provincial Health Services Authority (PHSA), will perform an initial health and mental health assessment within 48 h of intake. This assessment includes screening for physical health conditions, mental health issues, and substance use disorders (BCMHSUS, 2025). If a patient was previously diagnosed with cancer in the community, the CHS team can access an existing healthcare record of diagnosis and treatment. Participants noted that having an established healthcare record of a cancer diagnosis served as tangible proof, enabling individuals to face fewer barriers to care because their need for services was already documented and acknowledged.

In some instances, individuals detained in provincial institutions in BC were residents of another province, and in this case, the correctional healthcare team would have to wait for out-of-province records to proceed with referral and begin treatment:

Sometimes, there are delays in getting referrals. If they [patients] were not seen previously by BC Cancer and were seen out of province… that would create a bit of a delay for a lot of our patients, [as] we would need those records in order to do the referral.

Individuals who developed cancer symptoms while incarcerated or whose symptoms had not yet been diagnosed at the time of incarceration faced a more complex and precarious path to care. The key difference between these pathways lies in the burden placed on individuals who must self-report symptoms and advocate for their health needs in a system underpinned by mistrust and judgment. One nurse shared that the most significant barriers to care occurred in the early stages of illness: “Usually, once there's a set diagnosis, I don't think we are going to have as much resistance [from the healthcare team]. I just feel that it's the early stages that are the hardest because the list to see a doctor is really long.” In addition, the participant clarified: “I think it's easier once there is something written down… I feel once there is a diagnosis, the problem goes away as far as the healthcare…and corrections…are putting barriers.” These quotes emphasize that without a confirmed diagnosis and with limited access to a primary care provider, patients sometimes face mistrust, and their symptoms can be minimized or overlooked, resulting in delayed access to care.

Participants shared that people experiencing undiagnosed cancer symptoms that are not immediately apparent often face the additional challenge of overcoming stigma, needing to demonstrate that they are not seeking attention or medication but are genuinely in need of care. A participant highlighted: “The nurses may not take the inmate seriously, or they [patients] will put in so many requests that they're kind of ignored.” However, nurses who had previous experience of working in prisons reported navigating a delicate balance between responding to a valid health concern while being mindful of medication-seeking behaviors within the carceral environment. For example, individuals may resort to “cheeking” - hiding medication inside the mouth without swallowing -for selling or trading to others. As a result, participants described how patients seeking health assessments in provincial correctional institutions can face doubt about the credibility of their health concerns and experience an undue burden of proof.

The burden of proof refers to the effort required from a patient in prison to be taken seriously and how it must begin early in the care process, starting with how individuals initiate contact with health services. While the study focused on cancer, participants described this process more broadly in relation to a range of health needs. Participants shared how people detained in a provincial facility can ask to see a nurse or a doctor by communicating this request to Correctional officers and completing a health request form. The request is triaged, and a healthcare appointment is scheduled. While this seems a simple process, navigating it is often complicated by structural barriers and deeply rooted systemic inequities that manifest as personal challenges. For example, nurses shared their experience of seeing how patients who wanted to schedule an appointment with a healthcare team member sometimes had to recruit the support of a Correctional officer to complete a health request form if they lacked health literacy. “They [people in prison] have to fill out health requests while they are on their unit and then submit it by paper, and that's a big barrier because sometimes they would have to get a guard to write it out if they didn't have actual literacy.” As a result, the participants noted how the health request forms could lack specificity and detail, leading to a lower triage priority. For some, the dynamics in which individuals are making requests are shaped and compounded by histories of trauma, mental illness and institutional mistrust, where disclosing personal health concerns to staff who are not part of the healthcare team can feel unsafe: “They [patients] may not want to tell the guard their health problems, so sometimes the requests would be quite vague, and if things are really busy, they would be a low priority.” In addition, participants suggested that previous negative experiences with healthcare and correctional systems might make patients hesitant to report symptoms, fearing repercussions or being ignored. One nurse shared: “If they [patients] got in trouble for voicing concerns, they might not voice something, even if they did know something was wrong.” Finally, participants emphasized how the need for incarcerated individuals to self-report sensitive health information to correctional staff and earn the trust of the healthcare team places an unequal burden on people in prison, especially in high-risk and disempowering environments.

### Tensions and Negotiations in Cancer Care

The emphasis on security in a carceral environment follows people who experience incarceration into outpatient clinics and sometimes takes priority over the provision of cancer care. Nurses in our study described this situation as leading to tensions and negotiations within themselves as professionals, with institutional policies, with correctional staff, and sometimes with patients, to fulfill their professional obligations and provide timely, safe, and high-quality cancer care. Nurses described making frequent “accommodations” and “compromises” in their daily practices to uphold key nursing clinical values such as dignity, confidentiality and respect.

#### Privacy, Confidentiality and Transparency

One example of tensions and negotiations is the challenge of upholding patients’ rights to privacy, confidentiality, and transparency, as noted by the nurses in the study.

In most clinical encounters, patient privacy and confidentiality are key to providing safe care and fostering a rapport. However, medical appointments for cancer patients who experience incarceration are shaped by an inherent tension between patients’ confidentiality and the broader security protocols. Nurses noted that privacy was compromised by the presence of security officers, institutional clothing worn by patients and handcuffs. When patients visit a healthcare clinic in a correctional institution or are brought in for cancer treatments to community clinics, correctional officers are positioned outside an open door during private health conversations. Even when a nurse takes steps to protect a patient's health information, there is an awareness that this gesture may be more symbolic than real. As one nurse reflected, “There is a certain performance of protecting that privacy, knowing that everything else, every other component of the person's life, is not private.” A few nurses shared that this limitation to patients’ privacy in oncology outpatient clinics felt “difficult” and filled with “tension…because that is not the same dynamic as talking to someone from the general population, where you know privacy and confidentiality are enshrined as rights.” Nurses said they could “close a curtain, but couldn’t close the door, because the officers have to be outside the door.” Besides the physical presence of correctional officers who, one nurse reported, were often reluctant to hear patients’ sensitive health information, the interviews highlighted other highly visible and stigmatizing elements that compromised patients’ privacy and exposed their incarceration status in the waiting rooms of community clinics. These include shackles on arms and legs, and a bright red or orange jumpsuit worn by many patients. Only in a few instances did nurses encounter patients who were brought in unshackled and wearing street clothing, a decision potentially influenced by the patients’ security level. Participants shared that there is a “stigma as they [patients] are coming into an outpatient environment wearing institutional clothing, often legs and arms shackled,” portraying patients as “visibly institutionalized or visibly made vulnerable by those signifiers.” Participants experienced instances where patients declined cancer treatment appointments “because they didn’t want people to see them like that” and because it was “incredibly dehumanizing for them.” While participants could not speak more broadly about whether incarceration-related stigma affected timely access to cancer care, one nurse reflected that “patients’ own perception of their own identity can be very different.” The participant further elaborated: “We have a few [people from prisons] who do come in, and I see them in the inpatient or outpatient level coming in to see their doctors. I don't see them on the treatment floors.” Both inside and outside correctional settings, privacy or the lack of it, was shaped by material actors such as open doors, thin curtains, uniformed guards, and loud shackles.

#### Communications

Beyond the visible markers of incarceration discussed above, participants described how security concerns also influence more subtle aspects of the cancer care experience, including challenges in conducting pre-appointment assessments for people who experience incarceration. Typically, nurses phone new patients to complete intake assessments; however, it is “hard to do when they are incarcerated.” One nurse explained that while basic clinical information, such as diagnosis, medical history and medications can be obtained from the correctional healthcare team, mental health assessments and “how they [patients] are coping with their disease” were often missed. As the nurse described, the more personal aspects of assessment involve sensitive topics such as emotional well-being, levels of anxiety and suicidal ideations, which cannot be adequately explored by asking the medical providers. As a result, the participant described relying on in-person visits to complete these assessments, albeit in the presence of correctional officers.

#### Negotiations

Despite these constraints on providing cancer care, the interactions between nurses, patients and correctional officers in waiting rooms and clinical spaces, as described by nurses, reveal how care still occurs, often through subtle, relational, or material adaptations. For example, multiple participants reported prioritizing “get[ting] them [patients] into a room as soon as possible so that they are not sitting out in the waiting room.” For example, participants described drawing the curtains inside prison and community clinics to provide privacy across the threshold where correctional officers stood. When communicating follow-up care instructions to correctional officers, oncology nurses sometimes tried to “give them as much reasoning as possible without sharing any confidential pieces.” Finally, recognizing the potential emotional impact of receiving cancer care in a community setting, nurses created time and space for patients to express their feelings and made efforts to connect patients with counselors before or after appointments.

## Discussion

Findings from this study illustrate nurses’ views that the prison's security practices shape how, when, and whether cancer care is delivered. Drawing on interviews with nurses in British Columbia, Canada, and informed by ANT we generated the following insights: 1) institutional priorities, particularly those privileging confinement and control, often complicated the delivery of cancer care; 2) nurses perceived systemic stigma and institutional mistrust toward incarcerated patients, especially those without a formal cancer diagnosis; and 3) lack of confidentiality during healthcare encounters hindered patient-centred cancer care. Visible signs of incarceration, such as shackles and institutional clothing, intensified stigma, stripped patients of the usual protection of privacy afforded to others in community clinics and impacted care engagement. Despite this, within the security-centred environment that often positions healthcare as secondary, nurses frequently found ways to navigate professional tensions caused by dual responsibilities to patient care and security protocols.

These findings align with and extend prior work that documents systemic barriers to health services (Nyvoll, 2025) and cancer care for people who experience incarceration (Armes et al., 2024; Kouyoumdjian et al., 2017). Prisons have two distinct mandates: ensuring the custody of individuals and providing them with comparable standards of healthcare services available to the general population. Several authors argue that these goals are mutually exclusive and often undermine one another in practice (Nyvoll, 2025; Smith, 2000). Punishing people and caring for them remain fundamentally at odds (Nyvoll, 2025) because systems built to discipline rarely create the conditions for healing and wellness. Our finding that security protocols impact healthcare is echoed in a study from Norway (Nyvoll, 2025) and another from England (Armes et al., 2024). People in prison who seek health services often embody a dual identity as both patients and prisoners, which can lead to conflicting expectations and treatment (Nyvoll, 2025). For example, when patients report health concerns and seek care, they expect to receive treatment and restore their well-being (Nyvoll, 2025). However, people in prison are often viewed “as a criminal who was not to be trusted” (Nyvoll, 2025, p.11). Thus, when a prisoner becomes a patient, their identity as a prisoner is not erased, and the provision of care often reflects mistrust, stigma, and lack of health autonomy (Nyvoll, 2025). In this context, patients who seek medical attention frequently face diagnostic skepticism, where symptoms are scrutinized through a lens of suspicion (Armes et al., 2024; Lehrer, 2021). Armes et al. (2024) findings suggest that healthcare staff, constrained by limited time and resources, face difficulty distinguishing legitimate physical illness from issues related to mental health or substance use. Patients are often disbelieved not solely due to clinician unwillingness but as a result of systemic pressures and the perceived risk of “malingering” (Armes et al., 2024, p. 5). Consequently, the onus is placed on individuals to prove that their symptoms are genuine and warrant further investigation. This burden of proof is especially concerning for those presenting with vague or nonspecific symptoms and can result in harmful delays or dismissals. These relational dynamics can also be considered within a broader interdisciplinary scholarship that illuminates how carceral systems produce structural and psychological harms. For instance, public health scholars in the US observed how mass incarceration, shaped by systemic violence and institutional control, exacerbates health inequities (Cloud et al., 2023), while carceral psychology research has demonstrated how confinement, surveillance, and deprivation constrain autonomy and negatively affect mental health (Haney, 2002).

Providing cancer care to people who experience incarceration is intricate and tangled (Armes et al., 2024). For nurses in our study, it often led to ethical tensions between clinical values and security protocols. Specifically, privacy and confidentiality during healthcare encounters were routinely compromised for people in prison and when they attended cancer treatment appointments in outpatient clinics. As a result, patients may withhold information to avoid judgment, leading to incomplete assessments and making it difficult to develop trusting therapeutic relationships.

While our findings align with concerns shared by several authors who examined health and cancer care in prisons (Armes et al., 2024; Kouyoumdjian et al., 2017; Nyvoll, 2025), this study uniquely highlights how nurses navigate and negotiate tensions when providing cancer care for people who experience incarceration to uphold ethical nursing values. Specifically, nurses described actively “working around” institutional practices, such as drawing curtains to provide privacy, expediting patients’ admission in community clinics to reduce discomfort, and altering the process of medication administration during lockdowns. These approaches used by nurses illustrate how care providers, correctional staff, and artifacts such as curtains, doors, and schedules can either reinforce rigid carceral rules or counteract procedural and material barriers.

Applying a lens using the ANT helped reveal how both human (e.g., officers, nurses) and nonhuman actors (e.g., doors, curtains, schedules) shape cancer care delivery, producing both barriers and opportunities, sometimes reinforcing carceral control and sometimes enabling creative acts of patient-centred care. The delivery of cancer care for people in provincial institutions is not simply a medical endeavor but a complex and shifting interaction between correctional protocols, physical environments, and professional responsibilities. This perspective allows to view cancer care in a correctional context not only as a site of challenge but as an opportunity for reimagining care models and supporting patients’ dignity.

The findings from this study reinforce the need for trauma-informed and equity-focused approaches to cancer care across institutional boundaries. Nurses need to be supported in providing safe, ethical care and ensuring patients’ confidentiality and dignity. Implications for practice include a coordinated cancer screening strategy for incarcerated populations, particularly for cancers with high prevalence; enhanced communication pathways between correctional health providers and community-based cancer care teams; and provision of basic dignity items such as street clothing for patients attending outpatient community cancer clinics. After or before appointments, patients can be offered an opportunity to debrief with a nurse, counselor or peer support worker.

## Limitations

The sample included oncology nurses and nurse leaders in British Columbia and while their perspectives provide an important angle, further research might involve people who experience incarceration to explore their perspectives on cancer care. The lack of patient voices reflects well-documented ethical and institutional barriers to research in correctional settings, including power asymmetries, institutional gatekeeping, and the risk of perceived or actual coercion for participants. Future studies could address this gap through post-release participatory designs, where people with lived experience shape research priorities and interpretation outside the immediate control of correctional institutions.

Additionally, our findings reflect a professional grounded perspective, as our sample may represent nurses who were more willing or available to discuss these issues, and their responses may have been influenced by professional identities or social desirability. Despite the limitations, the interview data offered unique nursing perspectives on an under-researched area of Canadian cancer care, particularly in relation to provincial correctional settings. As a means to facilitate transferability of the findings (as opposed to universalizing generalizability), we provided contextual details about the study setting to enable the reader to establish similarities or differences with their context.

## Conclusion

This study explored how nurses in British Columbia perceive and navigate the provision of cancer care services to people who experience incarceration. By deepening understanding of the correctional cancer care landscape through a nursing lens, the study aimed to contribute to ongoing efforts that address cancer care disparities for this equity-deserving population. The key findings revealed precarious networks of previously unexamined interactions between nurses, the institutional environment, patients, and other material actors (e.g., protocols, curtains, and medications), as well as their dynamic roles in shaping cancer care within and beyond the walls of provincial correctional institutions. Specifically, nurses’ interviews underscore how custodial practices focused on security, control, and punishment negatively impact patient-centred cancer care by deprioritizing medical needs, limiting patients’ privacy during clinical encounters and considering patients’ healthcare requests through the lens of stigma and mistrust. Despite complex carceral barriers to cancer services, nurses engaged in practices to provide dignity-affirming care. Paying greater attention to adaptations and compromises that nurses perform when caring for people who experience incarceration can inspire low-barrier, supportive interventions and enhancements to existing care. Although these findings are particularly relevant to the BC context, they may also apply to similar settings in other provincial correctional systems in Canada, where healthcare is delivered separately from correctional services.

## Appendix A: Participants’ Demographics

**Table:**

Characteristic	Category	Number of Participants
*Professional Roles*	Front-line nurses (RN, LPN)	5
	Clinical nurse leaders / specialists (CNS, nurse leads, coordinators)	4
	Senior clinical administrators	2
*Practice Context*	Previously worked in correctional setting	6
	Community-based only (no correctional experience)	5
*Extent of involvement with patients who experience incarceration*	Recurring involvement	3
	Several encounters	5
	Single or no direct encounters	3

## Appendix B: Themes and Exemplar Quotes

**Table:**

Theme	Sub-Theme	Exemplar Quote
Security as the structuring logic of cancer care	X	“The safety and security of the prison is number one. The corrections officers run the show…”
Burden of proof and complex pathways to referral	X	“I feel once there is a diagnosis, the problem goes away as far as the healthcare…and corrections…are putting barriers.”
Tensions and negotiations in cancer care	privacy, confidentiality and transparency	“There is a certain performance of protecting that privacy, knowing that everything else, every other component of the person's life, is not private.”
	communications	Typically, nurses phone new patients to complete intake assessments; however, it is “hard to do when they are incarcerated.”
	negotiations	Multiple participants reported prioritizing “get[ting] them [patients] into a room as soon as possible so that they are not sitting out in the waiting room.”
